# Prognostic value of preoperative prognostic nutritional index and its associations with systemic inflammatory response markers in patients with stage III colon cancer

**DOI:** 10.1186/s40880-017-0260-1

**Published:** 2017-12-21

**Authors:** Jianhong Peng, Rongxin Zhang, Yixin Zhao, Xiaojun Wu, Gong Chen, Desen Wan, Zhenhai Lu, Zhizhong Pan

**Affiliations:** Department of Colorectal Surgery, State Key Laboratory of Oncology in South China, Collaborative Innovation Center for Cancer Medicine, Sun Yat-sen University Cancer Center, 651 Dongfeng Road East, Guangzhou, Guangdong 510060 P. R. China

**Keywords:** Prognostic nutritional index, Colon cancer, Systemic inflammatory response marker, Prognosis

## Abstract

**Background:**

The prognostic nutritional index (PNI) has been widely applied for predicting survival outcomes of patients with various malignant tumors. Although a low PNI predicts poor prognosis in patients with colorectal cancer after tumor resection, the prognostic value remains unknown in patients with stage III colon cancer undergoing curative tumor resection followed by adjuvant chemotherapy. This study aimed to investigate the prognostic value of PNI in patients with stage III colon cancer.

**Methods:**

Medical records of 274 consecutive patients with stage III colon cancer undergoing curative tumor resection followed by adjuvant chemotherapy with oxaliplatin and capecitabine between December 2007 and December 2013 were reviewed. The optimal PNI cutoff value was determined using receiver operating characteristic (ROC) curve analysis. The associations of PNI with systemic inflammatory response markers, including lymphocyte-to-monocyte ratio (LMR), neutrophil-to-lymphocyte ratio (NLR), platelet-to-lymphocyte ratio (PLR), and C-reactive protein (CRP) level, and clinicopathologic characteristics were assessed using the Chi square or Fisher’s exact test. Correlation analysis was performed using Spearman’s correlation coefficient. Disease-free survival (DFS) and overall survival (OS) stratified by PNI were analyzed using Kaplan–Meier method and log-rank test, and prognostic factors were identified by Cox regression analyses.

**Results:**

The preoperative PNI was positively correlated with LMR (*r* = 0.483, *P* < 0.001) and negatively correlated with NLR (*r* = − 0.441, *P* < 0.001), PLR (*r* = − 0.607, *P* < 0.001), and CRP level (*r* = − 0.333, *P* < 0.001). A low PNI (≤ 49.22) was significantly associated with short OS and DFS in patients with stage IIIC colon cancer but not in patients with stage IIIA/IIIB colon cancer. In addition, patients with a low PNI achieved a longer OS and DFS after being treated with 6–8 cycles of adjuvant chemotherapy than did those with < 6 cycles. Multivariate analyses revealed that PNI was independently associated with DFS (hazard ratios 2.001; 95% confidence interval 1.157–3.462; *P* = 0.013).

**Conclusion:**

The present study identified preoperative PNI as a valuable predictor for survival outcomes in patients with stage III colon cancer receiving curative tumor resection followed by adjuvant chemotherapy.

## Background

Colon cancer is a leading cancer and a leading cause of cancer-related deaths both in China [1] and worldwide [2]. Previous reports have shown that approximately one-third of all patients developed stage III or node-positive disease at diagnosis [3, 4]. Curative tumor resection is currently the mainstay treatment of non-metastatic colon cancer. A decade ago, the combined regimen of oxaliplatin and 5-fluorouracil was recommended as the standard postoperative therapy for stage III colon cancer on the basis of reported reduction in the risk of recurrence and death within the first 6 years after treatment [5, 6]. However, in previous studies, one-fourth to one-third of patients with stage III colon cancer would develop distant metastases despite receiving curative-intent treatment [7, 8]. Therefore, it is necessary to identify prognostic factors and individualize postoperative therapy according to patient classification.

Presently, survival outcomes of stage III colon cancer patients are mainly predicted with tumor-associated factors, such as tumor-node-metastasis (TNM) stage, tumor grade, tumor location, and carcinoembryonic antigen (CEA) level [9–11]. The survival is also determined by host-related factors, in particular, preoperative nutritional status and systemic inflammatory response (SIR) [12–14]. Accordingly, immunonutritional biomarkers are needed to further refine the staging system beyond tumor-associated factors that have been readily implemented into clinical treatment. Specifically, the SIR level of patients with various types of cancer is generally reflected by lymphocyte-to-monocyte ratio (LMR), neutrophil-to-lymphocyte ratio (NLR), platelet-to-lymphocyte ratio (PLR), and C-reactive protein (CRP) levels, which are also called SIR markers [15, 16]. The prognostic nutritional index (PNI), which was first proposed by Onodera et al. [17], is a prognostic index that is reflective of both the nutritional and immunological statuses, and it is determined using the serum albumin level and the lymphocyte count in peripheral blood. Recently, studies have demonstrated that a low PNI predicted poor survival outcomes of patients with different types of malignant tumors originating from the digestive system [18–20]. To date, the prognostic value of PNI in patients with stage III colon cancer undergoing curative tumor resection followed by adjuvant chemotherapy remains unknown. The present National Comprehensive Cancer Network (NCCN) guidelines recommend the administration of the fixed 6-month adjuvant chemotherapy for the patients with stage III colon cancer [21]. A favorable preoperative immunonutritional status was associated with good tumor response to chemotherapy [22, 23]. The necessity of full-course of adjuvant chemotherapy for stage III colon cancer might be questioned for the patients with a favorable immunonutritional status.

Therefore, the present study aimed to explore the prognostic value of PNI and its relationship with SIR markers and survival benefit of full-course adjuvant chemotherapy in patients with stage III colon cancer, in order to help stratify different risk subgroups and optimize the duration of adjuvant chemotherapy.

## Patients and methods

### Patient selection

We obtained the records of consecutive patients with stage III colon cancer who underwent tumor resection followed by adjuvant chemotherapy between December 2007 and December 2013 at Sun Yat-sen University Cancer Center, Guangzhou, China. All cases were staged according to the 2010 American Joint Committee on Cancer (AJCC) staging system. The inclusion criteria were as follows: (1) histologically confirmed adenocarcinoma; (2) curative resection of colon cancer; (3) XELOX adjuvant chemotherapy (oxaliplatin 130 mg/m^2^ administered intravenously on day 1 and capecitabine 1000 mg/m^2^ administered orally twice daily on days 1–14 for a 3-week cycle); (4) complete record of preoperative lymphocyte count and serum albumin level; (5) no anti-cancer therapy before tumor resection; and (6) at least 3-month postoperative follow-up. The present study was performed according to the ethical standards of the World Medical Association Declaration of Helsinki and was approved by the Institutional Review Board and Independent Ethics Committees of Sun Yat-sen University Cancer Center.

### Calculation of laboratory data

Laboratory data, including serum albumin and C-reactive protein levels, and blood cell counts, were collected from records of blood routine tests performed within 7 days before surgery. PNI was calculated using the following formula: PNI = serum albumin level (g/L) + 5 × total lymphocyte count (/L) [17]. LMR was calculated by dividing the total lymphocyte count by total monocyte count. Likewise, NLR and PLR were calculated by dividing the total neutrophil and platelet count, respectively, by total lymphocyte count.

### Follow-up

Physical examination, blood tests of CEA and carbohydrate antigen 19–9 (CA199), abdominal ultrasonography, and chest radiography were conducted every 3 months postoperatively. Chest/abdominal/pelvic computed tomography (CT) and colonoscopy were performed annually. Overall survival (OS) was defined as the interval from the date of surgery until death of any cause or the last follow-up; disease-free survival (DFS) was defined as the interval from surgery to disease recurrence, death, or the last follow-up. Patients without any event (metastasis or death) at the last follow-up date were regarded as random censoring. The last follow-up visit was in December 2016.

### Statistical analysis

The optimal PNI cutoff value for the prediction of 3-year OS was determined using the Cutoff Finder software program, which is an R software-engineered web-based system (http://molpath.charite.de/cutoff/), with receiver operating characteristic (ROC) curve analysis according to the highest Youden index. Categorical variables were compared using the Chi square or Fisher’s exact test. Continuous variables were compared using Student’s *t* test or the Mann–Whitney *U* test. The correlations of PNI with SIR markers were assessed using Spearman’s correlation analysis. Survival outcomes of patients with high and low PNI were compared using Kaplan–Meier estimator and log-rank test. Potential effects of clinical variables on DFS and OS were examined using univariate Cox proportional hazards analyses. Variables that were statistically significant with a *P* < 0.05 in the univariate Cox model were further assessed with a multivariate Cox model using a forward stepwise method. Hazard ratios (HRs) and 95% confidence intervals (CIs) were calculated. All analyses were performed using IBM SPSS statistics software, version 21.0 (IBM Corp., Armonk, NY, USA). All statistical tests used in this study were two-sided, and a *P* value < 0.05 was considered significant.

## Results

### Patient characteristics

Clinical records of 321 consecutive patients were reviewed, and 47 patients were excluded: 36 underwent adjuvant chemotherapy with other regimens, 5 were lost to follow-up, 4 underwent palliative tumor resection, and 2 had no preoperative blood assessment data. Of the 274 eligible patients, 156 (56.9%) were male and 118 (43.1%) were female, with a median age of 55 years (range 22–85 years); 207 (75.5%) had well/moderately differentiated tumors. The median number of metastatic lymph nodes was 2 (range 1–23). With regard to the AJCC stage, 10 (3.6%) patients were diagnosed with stage IIIA colon cancer, 202 (73.7%) with stage IIIB colon cancer, and 62 (22.6%) with stage IIIC colon cancer (Table 1).

**Table 1:** Clinicopathologic characteristics, preoperative systemic inflammatory response markers, and postoperative metastatic pattern of patients with stage III colon cancer

Variable	Overall [cases (%)]	High PNI group [cases (%)]	Low PNI group [cases (%)]	*P* value
Total	274	152 (55.5)	122 (44.5)	
Age (years)				
≤ 60	189 (69.0)	112 (73.7)	77 (63.1)	0.061
> 60	85 (31.0)	40 (26.3)	45 (36.9)	
Sex				0.274
Male	156 (56.9)	91 (59.9)	65 (46.7)	
Female	118 (43.1)	61 (40.1)	57 (53.3)	
Baseline BMI (kg/m^2^)				0.154
< 18.5	28 (10.2)	11 (7.2)	17 (13.9)	
18.5–25.0	187 (68.2)	105 (69.1)	82 (67.2)	
> 25.0	59 (21.5)	36 (23.7)	23 (18.9)	
Hemoglobin (g/L)				< 0.001
< 90	42 (15.3)	11 (7.2)	31 (25.4)	
≥ 90	232 (84.7)	141 (92.8)	91 (74.6)	
Tumor location				< 0.001
Right-sided colon	103 (37.6)	44 (28.9)	59 (48.4)	
Left-sided colon	171 (62.4)	108 (71.1)	63 (51.6)	
Tumor size (cm)				< 0.001
≤ 4	140 (51.1)	91 (59.9)	49 (40.2)	
> 4	134 (48.9)	61 (40.1)	73 (59.8)	
Differentiation				0.962
Well/moderate	207 (75.5)	115 (75.7)	92 (75.4)	
Poor/undifferentiated	67 (24.5)	37 (24.3)	30 (24.6)	
T stage				0.754
T1–T2	12 (4.4)	6 (3.9)	6 (4.9)	
T3	121 (44.2)	70 (46.1)	51 (41.8)	
T4	141 (51.5)	76 (50.0)	65 (53.3)	
N stage				0.762
N1	189 (69.0)	106 (69.7)	83 (68.0)	
N2	85 (31.0)	46 (30.3)	39 (32.0)	
TNM stage				0.290
IIIA	10 (3.6)	6 (3.9)	4 (3.3)	
IIIB	202 (73.7)	117 (77)	85 (69.7)	
IIIC	62 (22.6)	29 (19.1)	33 (27.0)	
Preoperative serum CEA level (ng/mL)				0.031
≤ 5	159 (58.0)	97 (63.8)	62 (50.8)	
> 5	115 (42.0)	55 (36.2)	60 (49.2)	
Cycles of XELOX adjuvant chemotherapy^a^				0.296
< 6	53 (19.3)	26 (17.1)	27 (22.1)	
6–8	221 (80.7)	126 (82.9)	95 (77.9)	
LMR				
≤ 4.00	144 (52.6)	59 (38.8)	85 (69.7)	< 0.001
> 4.00	130 (47.4)	93 (61.2)	37 (30.3)	
NLR				
≤ 2.05	134 (48.9)	90 (59.2)	44 (36.1)	< 0.001
> 2.05	140 (51.1)	62 (40.8)	78 (63.9)	
PLR				
≤ 142.99	134 (48.9)	102 (67.1)	32 (26.2)	< 0.001
> 142.99	140 (51.1)	50 (32.9)	90 (73.8)	
Postoperative metastasis^b^	53 (19.3)	22 (14.5)	31 (25.4)	0.024
Liver metastasis	21 (7.7)	9 (5.9)	12 (9.8)	0.231
Lung metastasis	12 (4.4)	5 (3.3)	7 (5.7)	0.331
Abdominopelvic metastasis	16 (5.9)	5 (3.3)	11 (9.0)	0.053

*PNI* prognostic nutritional index, *BMI* body mass index, *TNM stage* clinical tumor-node-metastasis stage, *CEA* carcinoembryonic antigen, *LMR* lymphocyte-to-monocyte ratio, *NLR* neutrophil-to-lymphocyte ratio, *PLR* platelet-to-lymphocyte ratio

^a^XELOX adjuvant chemotherapy was administrated as follow: oxaliplatin 130 mg/m^2^ administered intravenously on day 1 and capecitabine 1000 mg/m^2^ administered orally twice daily on days 1–14 for a 3-week cycle

^b^The metastatic sites of 4 patients were not recorded

### Cutoff values of PNI and SIR markers

**Fig. 1:**
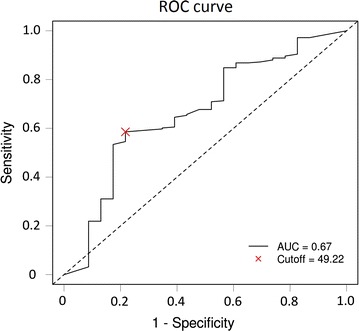
Receiver operating characteristic (ROC) curve analysis of preoperative prognostic nutritional index (PNI) for the prediction of 3-year overall survival (OS). The dashed line from the left bottom to the top right corners represents a random guess regardless of the positive and negative base rates. *AUC* area under the ROC curve

ROC curve analysis indicated that the optimal PNI cutoff value was 49.22, at the highest Youden index 0.37, with a sensitivity of 58.6% and specificity of 78.3% [area under the ROC curve (AUC), 0.67; 95% CI 0.55–0.79; *P* = 0.008; Fig. 1]. Patients were divided into the high PNI group [PNI > 49.22; *n* = 152 (55.5%)] and the low PNI group [PNI ≤ 49.22; *n* = 122 (45.5%)]. The median values of LMR, NLR, and PLR were 4.00 (range 0.20–16.00), 2.05 (range 0.75–12.36), and 142.99 (range 51.78–830.00), respectively, which were used as cutoff values.

### Relationships of PNI with clinicopathologic characteristics and SIR markers

The median age of the low PNI group was 58 years (range 27–85 years), which was significantly older than that of the high PNI group (median age 53 years; range 22–79 years; *P* = 0.003). The relationships between PNI and clinicopathologic characteristics are shown in Table 1. A low PNI was associated with low preoperative hemoglobin levels (*P* < 0.001), right-sided colon tumors (*P* < 0.001), large tumor size (*P* < 0.001), and high CEA levels (*P* = 0.031). No significant association was noted between PNI and other clinicopathologic characteristics.

**Fig. 2:**
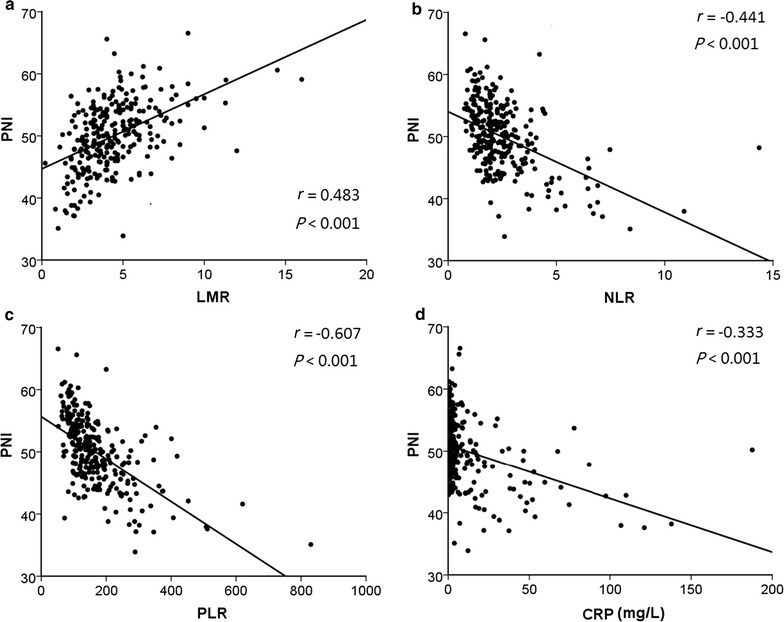
Correlations of the prognostic nutritional index (PNI) with systemic inflammatory response (SIR) markers in all patients with stage III colon cancer. Correlations between PNI and (**a**) lymphocyte-to-monocyte ratio (LMR), (**b**) neutrophil-to-lymphocyte ratio (NLR), (**c**) platelet-to-lymphocyte ratio (PLR), and (**d**) C-reactive protein (CRP) level were assessed using Spearman’s correlation analysis

Patients with low PNI presented a significantly lower median lymphocyte count (1.40 × 10^9^/L vs. 2.00 × 10^9^/L, *P* < 0.001) but higher median CRP level (4.23 mg/L vs. 2.15 mg/L, *P* < 0.001) compared with those with high PNI. The relationships between PNI and SIR markers among all patients are shown in Table 1. A low PNI was associated with a low LMR (*P* < 0.001), a high NLR (*P* < 0.001), and a high PLR (*P* < 0.001). PNI was found to be positively correlated with LMR (*r* = 0.483, *P* < 0.001, Fig. 2a) but negatively correlated with NLR (*r* = − 0.441, *P* < 0.001, Fig. 2b), PLR (*r* = − 0.607, *P* < 0.001, Fig. 2c), and CRP level (*r* = − 0.333, *P* < 0.001, Fig. 2d).

### Association between PNI and survival outcome

Follow-up data of all included patients were available for analysis. The median follow-up was 46 months (range 3–74 months). During follow-up, 55 (20.1%) patients experienced tumor recurrence, and finally, 30 (10.9%) died of tumor progression. With regard to the entire study population, the 3-year DFS and OS rates were 81.8% and 91.4%.

**Fig. 3:**
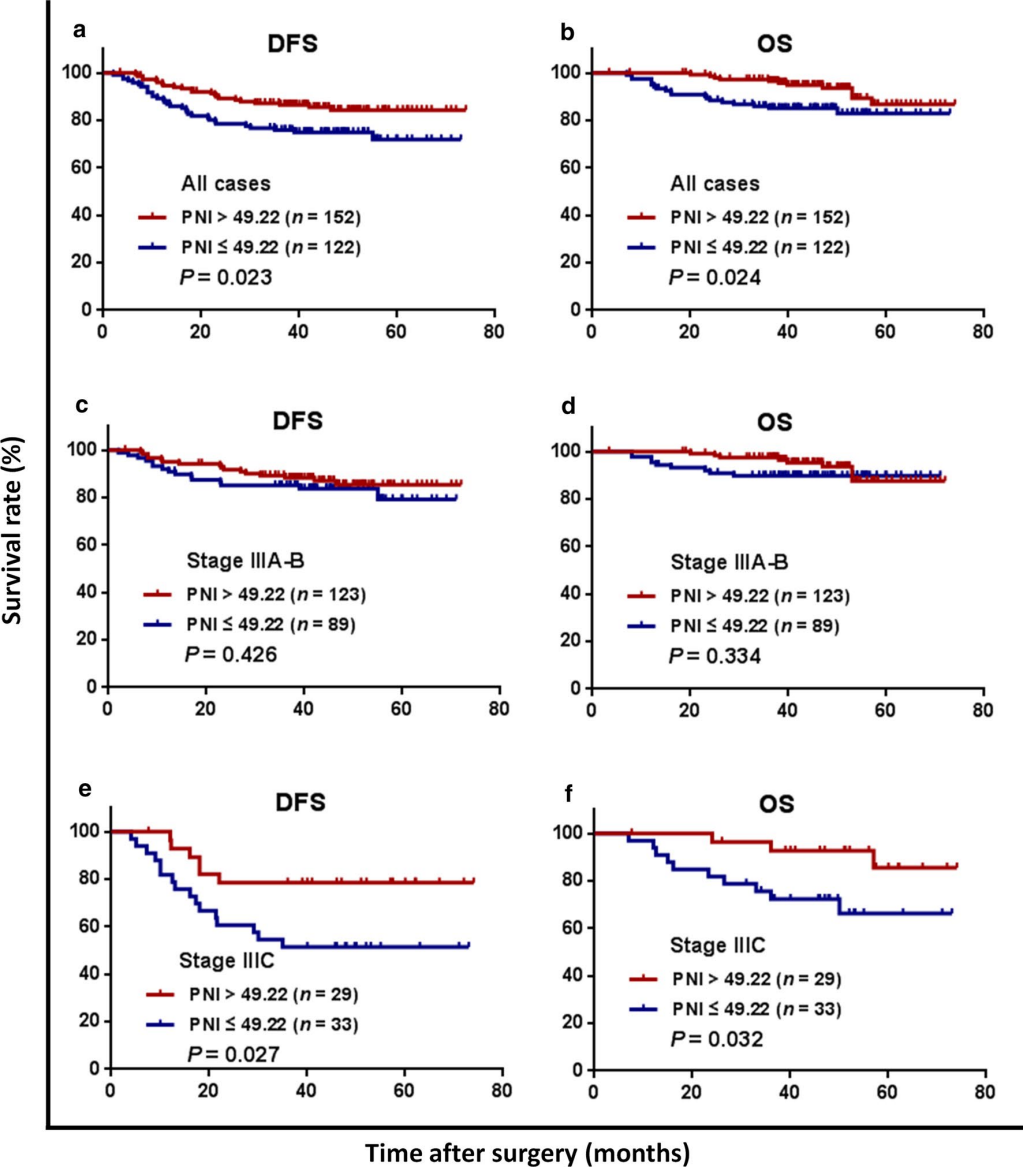
Kaplan–Meier survival curves of patients with stage III colon cancer grouped by prognostic nutritional index (PNI) and stratified by clinical stage. **a** The disease-free survival (DFS) curves of all patients; **b** the overall survival (OS) curves of all patients; **c** the DFS curves of patients with stage IIIA–B colon cancer; **d** the OS curves of patients with stage IIIA–B colon cancer; **e** the DFS curves of patients with stage IIIC colon cancer; **f** the OS curves of patients with stage IIIC colon cancer. XELOX adjuvant chemotherapy was administrated as follow: oxaliplatin 130 mg/m^2^ administered intravenously on day 1 and capecitabine 1000 mg/m^2^ administered orally twice daily on days 1–14 for a 3-week cycle

The relationships between PNI and postoperative metastases are also shown in Table 1. Postoperative metastasis was more common in the low PNI group than in the high PNI group (25.4% vs. 14.5%, *P* = 0.024). In contrast, the metastatic sites were not significantly different between the two groups (all *P* > 0.05). The 3-year DFS and OS rates were significantly lower in the low PNI group than in the high PNI group (DFS: 75.9% vs. 86.5%, *P* = 0.023, Fig. 3a; OS: 85.1% vs. 96.6%, *P* = 0.024, Fig. 3b). There were no differences in the 3-year DFS and OS rates among patients with stage IIIA–B colon cancer between the low and high PNI groups (DFS: 85.2% vs. 86.5%, *P* = 0.426, Fig. 3c; OS: 89.8% vs. 97.5%, *P* = 0.334, Fig. 3d). However, among patients with stage IIIC colon cancer, the 3-year DFS and OS rates were significantly lower in the low PNI group than in the high PNI group (DFS: 51.5% vs.78.6%, *P* = 0.027, Fig. 3e; OS: 72.3% vs. 92.7%, *P* = 0.032, Fig. 3f).

**Fig. 4:**
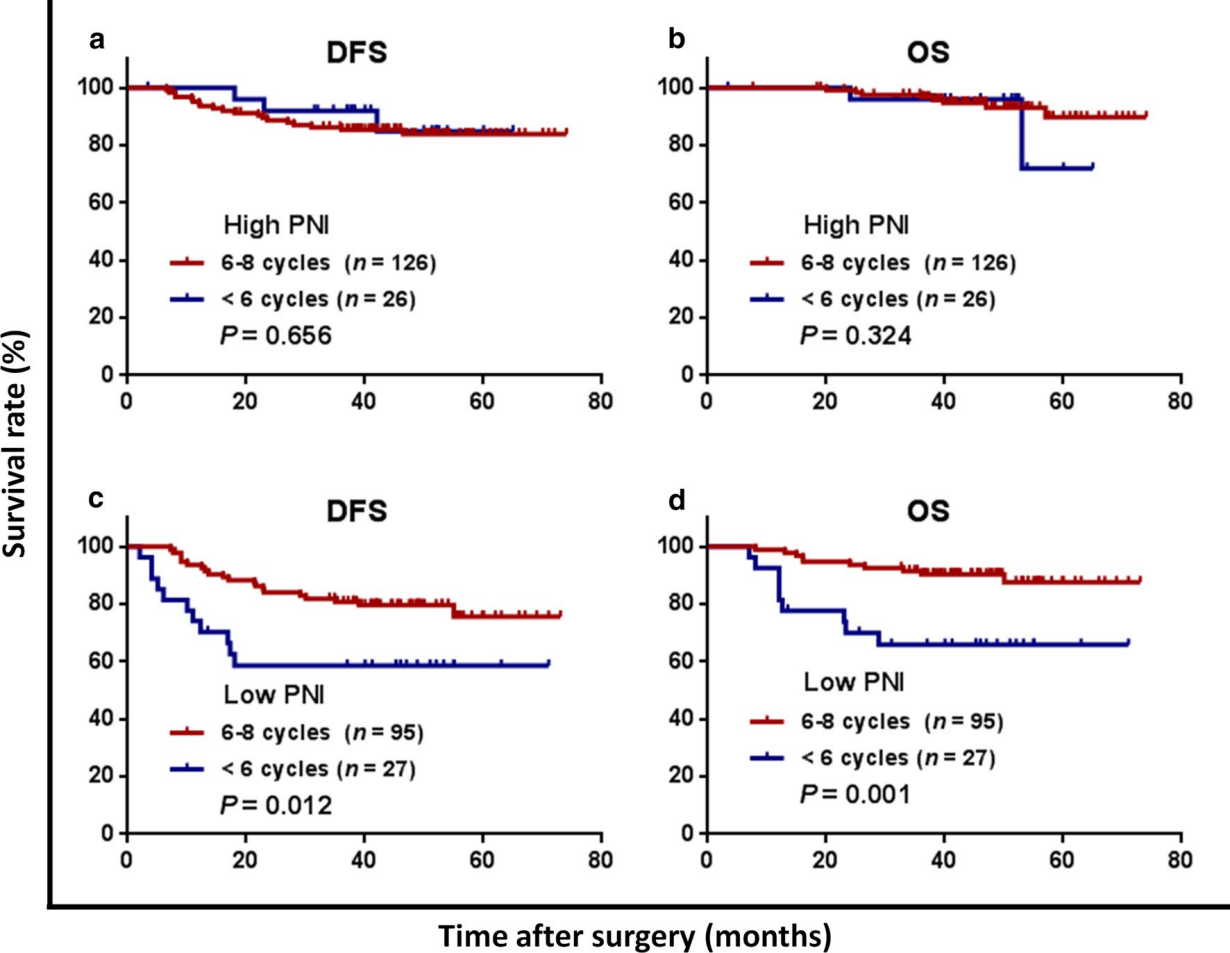
Kaplan–Meier curves of patients with stage III colon cancer in high and low PNI groups stratified by cycles of XELOX adjuvant chemotherapy. **a** The disease-free survival (DFS) curves in the high PNI group; **b** the overall survival (OS) curves in the high PNI group; **c** the DFS curves in the low PNI group; **d** the OS curves in the low PNI group

The association of adjuvant chemotherapy with survival was evaluated in both PNI groups. Among patients with a high PNI, the 3-year DFS and OS rates were comparable between those who received 6–8 cycles of XELOX adjuvant chemotherapy and those who received less than six cycles (DFS: 85.4% vs. 92.0%, *P* = 0.656, Fig. 4a; OS: 96.7% vs. 96.0%, *P* = 0.324, Fig. 4b). Among patients with a low PNI, the 3-year DFS and OS rates were significantly higher in patients who received 6–8 cycles of XELOX adjuvant chemotherapy than in those who received less than six cycles (DFS: 80.8% vs. 58.6%, *P* = 0.012, Fig. 4c; OS: 90.5% vs. 65.9%, *P* = 0.001, Fig. 4d).

### Prognostic analysis of clinical factors

Univariate analysis revealed that a low PNI (HR 1.889; 95% CI 1.094–3.262; *P* = 0.023) and male gender (HR 1.943; 95% CI 1.081–3.494; *P* = 0.026) were significant negative predictors for 3-year DFS; a low PNI (HR 2.353; 95% CI 1.118–4.950; *P* = 0.024) and right-sided colon tumor (HR 2.243; 95% CI 1.089–4.619; *P* = 0.028) were significant negative predictors for 3-year OS, whereas 6–8 cycles of adjuvant chemotherapy (HR 0.308; 95% CI 0.148–0.640; *P* = 0.002) was a significant protective factor for 3-year OS (Table 2).

**Table 2:** Univariate analyses of prognostic factors for disease-free survival and overall survival of patients with stage III colon cancer

Variable	Disease-free survival		Overall survival	
	HR (95% CI)	*P* value	HR (95% CI)	*P* value
Age (> 60 years vs. ≤ 60 years)	1.401 (0.804–2.443)	0.234	1.390 (0.661–2.925)	0.385
Sex (male vs. female)	1.943 (1.081–3.494)	0.026	1.902 (0.871–4.154)	0.107
BMI(< 18.5 kg/m^2^ vs. ≥ 18.5 kg/m^2^)	0.760 (0.274–2.107)	0.598	1.892 (0.724–4.944)	0.193
Hemoglobin (< 90 g/L vs. ≥ 90 g/L)	0.965 (0.455–2.047)	0.926	0.818 (0.285–2.346)	0.709
Tumor location (right-sided colon vs. left-sided colon)	1.356 (0.787–2.334)	0.272	2.243 (1.089–4.619)	0.028
Tumor size (> 4 cm vs. ≤ 4 cm)	0.941 (0.548–1.613)	0.824	1.046 (0.511–2.140)	0.902
Differentiation (poor vs. well/moderate)	1.143 (0.621–2.106)	0.667	1.181 (0.525–2.656)	0.687
T stage (T4 vs. T1–3)	1.553 (0.890–2.708)	0.121	1.709 (0.797–3.668)	0.169
N stage (N2 vs. N1)	1.573 (0.907–2.729)	0.107	1.487 (0.716–3.089)	0.287
CEA (> 5 ng/mL vs. ≤ 5 ng/mL)	1.557 (0.908–2.671)	0.107	0.987 (0.479–2.034)	0.972
Adjuvant chemotherapy (6–8 cycles vs. < 6 cycles)	0.590 (0.320–1.087)	0.091	0.308 (0.148–0.640)	0.002
LMR (> 4.00 vs. ≤ 4.00)	0.840 (0.488–1.447)	0.530	0.759 (0.365–1.578)	0.460
NLR (> 2.05 vs. ≤ 2.05)	0.915 (0.534–1.568)	0.746	0.872 (0.425–1.789)	0.708
PLR (> 142.99 vs. ≤ 142.99)	1.067 (0.622–1.831)	0.812	1.091 (0.533–2.237)	0.811
PNI (≤ 49.22 vs. > 49.22)	1.889 (1.094–3.262)	0.023	2.353 (1.118–4.950)	0.024

*HR* hazard ratio, *CI* confidence interval, *BMI* body mass index, *CEA* carcinoembryonic antigen, *LMR* lymphocyte-to-monocyte ratio, *NLR* neutrophil-to-lymphocyte ratio, *PLR* platelet-to-lymphocyte ratio, *PNI* prognostic nutritional index

In the multivariate Cox proportional hazards model, gender (HR 2.061; 95% CI 1.144–3.713; *P* = 0.016) and PNI (HR 2.001; 95% CI 1.157–3.462; *P* = 0.013) were identified as independent predictors of 3-year DFS, and the number of cycles of adjuvant chemotherapy was identified as an independent predictor of 3-year OS (HR 0.325; 95% CI 0.156–0.678; *P* = 0.003) (Table 3).

**Table 3:** Multivariate analyses of prognostic factors for disease-free survival and overall survival of patients with stage III colon cancer

Variable	Disease-free survival		Variable	Overall survival	
	HR (95% CI)	*P* value		HR (95% CI)	*P* value
Sex (male vs. female)	2.061 (1.144–3.713)	0.016	Adjuvant chemotherapy (6–8 cycles vs. < 6 cycles)	0.325 (0.156–0.678)	0.003
PNI (≤ 49.22 vs. > 49.22)	2.001 (1.157–3.462)	0.013	Tumor location (right-sided colon vs. left-sided colon)	1.909 (0.916–3.977)	0.084
			PNI (≤ 49.22 vs. > 49.22)	2.082 (0.978–4.429)	0.057

*HR* hazard ratio, *CI* confidence interval, *PNI* prognostic nutritional index

## Discussion

In the current study, we found that a low PNI was significantly associated with SIR markers and poor 3-year survival outcomes in stage III colon cancer patients who received curative surgery and adjuvant chemotherapy, especially in those with stage IIIC colon cancer. Furthermore, we found that only patients with PNI ≤ 49.22 who were treated with 6–8 cycles of XELOX adjuvant chemotherapy had longer survivals compared with those receiving less than six cycles. Moreover, PNI was demonstrated as an independent prognostic factor for 3-year DFS.

Several mechanisms could contribute to the compromise of long-term survival by a low PNI in patients with stage III colon cancer. First, it has been widely accepted that SIR is an important regulator of tumor cell growth, angiogenesis, migration, invasion, and metastasis through the recruitment of T lymphocytes, tumor-associated macrophages, and circulating cytokines [24, 25]. As a basic component of cellular immunity, a low lymphocyte count might be responsible for a poor and insufficient immunologic reaction against tumor progression. Iseki et al. [26] reported that patients with high peripheral lymphocyte count and percentage were likely to achieve a higher 5-year survival rate than those with low peripheral lymphocyte count and percentage. On the other hand, a decreased serum albumin level partially reflects a malnutritional status in the host and weak human systematic defense, and thus, might be associated with increased morbidity and mortality of cancer [27, 28]. Additionally, albumin has been identified as an important marker to aid in cancer control through stabilization of cell growth and DNA replication, and it serves as a versatile factor for multiple antioxidants and even protects against sex hormone-induced cancers [29]. In vivo, the absence of albumin was confirmed as being associated with a disturbed inflammatory response [30]. Previous studies have demonstrated that patients with decreased PNI may have an enhanced SIR, thus leading to a poor prognosis among cancer patients [31, 32], and these findings are consistent with our results. Therefore, it is quite understandable that an association of PNI with long-term prognosis was found in colon cancer patients.

Interestingly, we found that PNI was associated with OS and DFS in patients with stage III colon cancer, especially with stage IIIC colon cancer. This could be mainly attributed to the disparate characteristics between stage IIIC and stage IIIA–B colon cancer patients. Zhang et al. [31] have shown that PNI was closely associated with OS in patients with metastatic intrahepatic cholangiocarcinoma, but this association was not found in patients with local disease. In general, patients with advanced disease were more likely to undergo long-term severe nutritional consumption, and these patients subsequently experienced more severe weight and muscle loss. Additionally, these long-term severe nutritional consumption could easily lead to malnutrition and an aggravated inflammatory status [33]. Furthermore, patients with stage IIIC colon cancer might experience high tumor burden, which might promote the presence of a high amount of circulating inflammatory cytokines to maintain immunosuppression [34]. Therefore, the long-term survival of patients with stage IIIC colon cancer was more susceptible to the preoperative immunonutritional status.

Currently, available data recommends 6 months of oxaliplatin-based adjuvant chemotherapy as the routine postoperative therapy for stage III colon cancer patients [35]. Similarly, our present study found that 6–8 cycles of adjuvant chemotherapy was a protective factor of 3-year OS in total patients. However, we observed the different survival impact of adjuvant chemotherapy intensity among patients with either a high or low PNI. Patients with a high PNI did not benefit more from full-course (6–8 cycles) adjuvant chemotherapy compared with those receiving less than six cycles of XELOX adjuvant chemotherapy. However, for patients with a low PNI, full-course XELOX adjuvant chemotherapy was associated with higher 3-year DFS and OS rates. These findings might indicate that the malnutritional status may impair the efficacy of adjuvant chemotherapy. Previous data have shown that improved immune function reduced the recurrence rate and prolonged the survival of patients postoperatively [36, 37]. We consider that the high PNI group benefited from the combined anti-cancer impact of chemotherapy and enhanced immune function, and thus, no difference in survival was found between patients who received less than 6 and 6–8 cycles of adjuvant chemotherapy. It is necessary to pay more attention to assess the immunonutritional status for the better guidance of adjuvant chemotherapy. We consider that 6–8 cycles of XELOX adjuvant chemotherapy should be performed for patients with a low PNI to minimize the risk of tumor recurrence.

We found that a low PNI was also associated with old age, and this finding is consistent with the results of previous studies [38, 39]. The finding indicates that malnutrition and immunosuppression have become common problems in elderly patients who underwent surgery. In addition, the present study demonstrated that PNI was associated with aggressive clinicopathologic features of colon cancer patients, including a low hemoglobin level, right-sided colon tumor, large tumor size, and high CEA level. Moreover, PNI served as a predictor for advanced tumor depth, lymph node involvement, tumor undifferentiation, and poor TNM classification in colorectal cancer [40, 41]. These findings clearly showed that a low preoperative immunonutritional status indicated a high potential risk of tumor recurrence for colorectal cancer patients.

Previous studies have identified PNI as a valuable marker for predicting oncologic prognosis owing to several strengths, including high reliability, high reproducibility, and low cost [38, 42]. Therefore, it can be easily applied in clinical practice and serve as a valuable tool in prognostication for patients with colon cancer. Determining the preoperative PNI could provide useful information for the therapeutic decision-making. For the low PNI group, a feasible nutrition intervention before or during anti-cancer treatment was necessary. Nutrition support combined with anti-cancer treatment has been confirmed to prolong survival in advanced gastric cancer patients with a high risk of malnutrition [43]. Moreover, in a previous study, enteral nutrition increased the chance of chemotherapy completion that was planned in malnourished patients, which could further translate into survival prolongation [44]. In addition, PNI will be helpful for making clinical decisions on postoperative treatment in patients with stage III colon cancer. Once patients were diagnosed with stage IIIC disease with low PNI, we strongly recommend intensified or full-course adjuvant chemotherapy.

Unlike the multivariate analysis results of the present study, accumulating evidence supported that advanced T and N stages were associated with worse survival outcome in stage III colon cancer patients [45, 46]. In fact, the entire study population showed a favorable 3-year DFS and OS rates (81.8% and 91.4%) with up to 80% patients receiving at least 6 cycles of XELOX chemotherapy after curative tumor resection in the current study. A previous study has demonstrated that benefits of adjuvant chemotherapy did not vary based on N stage [47]. Therefore, the protective effect of adjuvant chemotherapy might overcome the survival impact of N or T stage. In addition, we found male and right-sided colon tumor were risk factors of short survival in patients with stage III colon cancer. A pooled analysis on the Adjuvant Colon Cancer End Points (ACCENT) database indicated that the male gender was associated with significantly inferior prognosis of early-stage colon cancer when compared with the female gender in terms of time to recurrence, DFS, and OS [48]. There may be broad underlying hormonal, genetic, or molecular mechanisms that predispose women to more favorable prognosis. Another prognostic analysis revealed that patients with stage III colon cancer located proximally had shorter survival than those with cancer located distally [9]. Symptoms of right-sided colon cancer are often lacking or unspecific, and specific symptoms (such as obstruction and bleeding) occur when the cancer is advanced. Moreover, gradual linear increases in CpG island methylator phenotype, microsatellite instability, and v-Raf murine sarcoma viral oncogene homolog B1 (*BRAF*) mutations from the rectum to the ascending colon indicated that tumors originating in the right-sided colon were less differentiated, more aggressive and advanced compared with those in the left-sided colon, which contributed to poor survival outcome in patients with right-sided colon cancer [49, 50].

Some limitations of the present study should be acknowledged. First, this retrospective study included an uncontrolled methodology and a limited number of patients from a single institution. Therefore, the findings need to be validated in a larger cohort of patients. Second, PNI was only assessed at a single time point before treatment, and its changes during anti-cancer therapy, which may have a more significant prognostic impact on patients, were not determined. Moreover, the 5-year survival data were unavailable owing to insufficient follow-up duration. This limitation may have led to underestimation of the PNI impact on OS. Nevertheless, we suggest that PNI should be considered in the routine evaluation of colon cancer patients before surgery.

## Conclusions

The present study revealed that PNI could serve as a simple and valuable marker for predicting long-term survival and SIR in patients with stage III colon cancer undergoing curative tumor resection followed by adjuvant chemotherapy. The preoperative PNI is suggested as an item of the routine assessment for colon cancer patients.
